# Opportunities for Policy Interventions to Reduce Youth Hookah Smoking in the United States

**DOI:** 10.5888/pcd9.120082

**Published:** 2012-11-15

**Authors:** Daniel S. Morris, Steven C. Fiala, Rebecca Pawlak

**Affiliations:** Author Affiliations: Steven C. Fiala, Rebecca Pawlak, Oregon Health Authority, Portland, Oregon.

## Abstract

Preventing youth smoking initiation is a priority for tobacco control programs, because most adult tobacco smokers become addicted during adolescence. Interventions that restrict the affordability, accessibility, and marketing of cigarettes have been effective in reducing youth cigarette smoking. However, increasing numbers of youth are smoking tobacco using hookahs. Predictors of smoking tobacco with hookahs are the same as those for smoking cigarettes. Established interventions that curb youth cigarette smoking should therefore be effective in reducing hookah use. Potential policy interventions include equalizing tobacco tax rates for all tobacco types, requiring warning labels on hookah tobacco and accurate labeling of product contents, extending the cigarette flavoring ban to hookah tobacco, enacting smoke-free air laws and removing exemptions for hookah lounges, and expanding shipping restrictions on tobacco products.

## Introduction

Preventing youth smoking is a priority for tobacco use control programs, because most tobacco users become addicted during adolescence (1). A range of public policies restricting the affordability, accessibility, and marketing of cigarettes has helped cut youth cigarette smoking nearly in half between 1998 and 2009 (2). Although fewer children and adolescents are smoking cigarettes, an increasing number are smoking tobacco using a hookah, also known as a waterpipe, nargile, goza, or hubble bubble (3). In 2011, 18.5% of 12th-grade students reported having smoked a hookah in the past year (4).

Hookahs are instruments used to smoke flavored tobacco, which is called *shisha*, or *maassel*. Shisha is a wet mixture of tobacco, sweetener, and flavorings. Shisha comes in various flavors, including strawberry, cappuccino, and cotton candy. A typical hookah has a fired-clay head that holds the shisha, a glass or acrylic bowl that is filled with water, and leather or plastic hoses through which users inhale the smoke. A metal screen or a piece of aluminum foil with holes punched in it covers the shisha, and a piece of burning charcoal is placed on top. When the smoker inhales through the hose, the smoke is drawn through the water, which cools the smoke.

Many hookah smokers believe that smoking a hookah carries less risk of tobacco-related disease than cigarette smoking (5). However, hookah smoke contains many of the same toxins as cigarette smoke and has been associated with lung cancer, respiratory illness, low birth weight, and periodontal disease (6). Studies of youth and young adults have found that predictors of smoking hookah are the same as those for smoking cigarettes, including social acceptability (7), having friends and family members who smoke (8), and perceiving that smoking a hookah is not harmful (3). Because predictors of cigarette and hookah smoking among youth are consistent, established interventions to reduce youth cigarette smoking should be effective for reducing hookah smoking. This article highlights potential policy interventions to reduce youth hookah use.

## Price

The single most powerful intervention to reduce youth smoking is raising the price of tobacco; adolescents and young adults are 2 to 3 times more price sensitive than adults (9). Hookahs are a type of pipe, and shisha, the flavored tobacco smoked in hookahs, is a variety of pipe tobacco. The federal tax on pipe tobacco is $2.83 per pound, which is nearly $22 per pound less than the tax on cigarette tobacco (10).

Equalizing the federal tax rates between loose pipe tobacco and loose cigarette tobacco would increase the price of shisha and likely reduce youth hookah smoking. In Oregon, a 50-gram package of shisha retails for about $3.65, which includes federal and Oregon state tobacco tax. Increasing the federal tax on loose pipe tobacco from $2.83 per pound to $24.78 per pound would add $2.42 in federal tax and at least $1.57 in Oregon state tax to a 50-gram package of shisha (Oregon’s 65% tax rate is applied to the wholesale price, which includes the federal tax). This would more than double the retail price of shisha. For every 10% increase in the real price of cigarettes, the number of youth who smoke drops by 6% to 7% (9). Assuming the same relationship holds for shisha, a 100% increase in price would lower youth consumption by at least 60%.

## Health Warnings

On the basis of research on youth cigarette smoking (11), youth who do not perceive hookah smoking as harmful are more likely to do it (3,8). Young people view hookah smoking as safer than smoking cigarettes and incorrectly believe that hookah smoke has less nicotine and is less toxic than cigarettes because of the filtering mechanism of the water in the base of the hookah (12). These findings suggest that an opportunity exists to reduce youth hookah use by educating youth about the adverse health effects associated with hookah smoking.

One way to educate youth about the dangers of tobacco use is to require warning labels on tobacco products and advertisements. Graphic warning labels are effective at raising awareness of the dangers of tobacco and increasing intentions to quit use (13). Federal law requires warning labels on cigarettes and smokeless tobacco but not on pipe tobacco. Warning labels on all tobacco products (including shisha) would inform consumers that hookah smoking is not safe. In addition to health warning labels, hookah tobacco products also need accurate product labeling. Some shisha brands have labels with incorrect information. One study (14) found that labeling of hookah tobacco did not reflect actual nicotine delivery to smokers; those who smoked a brand of hookah tobacco labeled 0.05% nicotine had greater plasma nicotine levels than those who smoked a brand of hookah tobacco labeled 0.5% nicotine. These inaccurate labels may perpetuate users’ beliefs that hookah tobacco is safer than other tobacco products (14).

## Regulation of Flavored Tobacco

Shisha is available in dozens of candy, fruit, coffee, and cocktail flavors. Flavors mask the harshness of tobacco and make it easier for new users to start using tobacco (15). The fruit flavors of hookah tobacco and the sweet smell of hookah smoke also contribute to the perception that hookah smoking is safer than cigarettes (12). The 2009 Family Smoking Prevention and Tobacco Control Act banned flavored cigarettes with the exception of menthol (16). However, the flavor ban does not extend to cigars, smokeless tobacco, or pipe tobacco. Extending the cigarette flavor ban to pipe tobacco would likely make hookah less appealing, particularly to youth. The Food and Drug Administration has the authority to extend a flavor ban to any tobacco product without an additional act of Congress (16). State and local governments can also pass laws banning the sale of flavored tobacco, which would further limit shisha sales.

## Smoke-Free Environments

As of December 2010, 25 states and the District of Columbia had comprehensive smoke-free laws prohibiting smoking in worksites, restaurants, and bars (17). Some states, including Oregon, have comprehensive smoke-free laws with exemptions that permit indoor smoking in cigar bars and smoke shops. Hookah lounges in Oregon are allowed to operate under the smoke shop exemption. Although three-fourths of the largest cities in the United States ban cigarette smoking in bars, hookah tobacco smoking may be permitted in nearly 90% of these cities via exemptions in clean indoor air laws that permit hookah smoking in smoke shops (18). The air inside hookah lounges contains high concentrations of secondhand smoke, creating hazardous conditions for patrons and employees (19). Oregon’s smoke-free law took effect in 2009; in the following 30 months, 30 applications for hookah lounges were submitted to the state (19). As of April 2012, 15 hookah lounges were operating in Oregon with certification to allow indoor smoking. Oregon Healthy Teens, Oregon’s youth behavioral risk factor survey, indicated that the prevalence of hookah use increased significantly from 2.7% in 2008 to 5.1% in 2009 among 8th-grade students in counties with hookah lounges. In counties without hookah lounges during the same time, the prevalence of hookah use by 8th-grade students increased from 1.6% to 1.9% (19).

In addition to protecting people from exposure to secondhand smoke, smoke-free laws help decrease the perception of smoking as an acceptable behavior (20), which in turn promotes cessation and discourages youth initiation of tobacco use. Conversely, the presence of hookah lounges creates and reinforces a community norm accepting of hookah smoking. Nearly 30% of high school students in San Diego learned about hookah smoking from seeing a hookah lounge, and current hookah users were more likely to know of a hookah lounge in their community (21). Furthermore, lounges reinforce pro-hookah messages in advertisements and on social networking sites. A content analysis of 144 hookah lounge websites across the United States found that only 4% included a tobacco-related warning on any page (22); the word “tobacco” appeared on 58% of the websites. Hookah lounge websites most commonly focused on flavorings, pleasure, relaxation, product quality, and the cultural and social aspects of hookah smoking; information on age limits, health warnings, and the involvement of tobacco in hookah smoking was limited (22). Smoke-free laws without exceptions for hookah lounges can protect more workers from secondhand smoke, limit hookah misperceptions perpetuated on hookah lounge websites, and create community norms that discourage hookah smoking.

## Internet and Mail-Order Access

Dozens of Internet sites sell shisha tobacco for home delivery. Online sales make it easier for youth to access tobacco. Major credit card companies agreed not to process online payments for cigarettes (23) but have not stopped processing payments for shisha. The Prevent All Cigarette Trafficking Act stops the US Postal Service from shipping cigarettes, roll-your-own tobacco, and smokeless tobacco but does not prohibit shipping shisha or other pipe tobacco products (24). Expanded restrictions on credit processing for Internet purchases and on shipping tobacco products would make hookah smoking less accessible to youth.

## Conclusion

Predictors of youth hookah smoking are similar to predictors of youth cigarette smoking. Therefore, successful strategies for reducing cigarette use among youth and young adults should also work for hookah use. There are many opportunities for policy interventions to reverse rising rates of youth hookah smoking. Equalizing tobacco tax rates would make hookah smoking less affordable for youth, and product warning labels would inform both youth and other users about the dangers of hookah smoking. Flavor bans for pipe tobacco may reduce hookah’s appeal to youth, and expanding shipping restrictions on tobacco products would make hookah smoking less accessible to youth. Smoke-free laws without exemptions would prevent hookah lounges from opening, and thereby decrease youth exposure to environments that normalize hookah smoking.
